# Evaluation of the efficacy and safety of lanreotide in combination with targeted therapies in patients with neuroendocrine tumours in clinical practice: a retrospective cross-sectional analysis

**DOI:** 10.1186/s12885-015-1512-6

**Published:** 2015-07-04

**Authors:** Jaume Capdevila, Isabel Sevilla, Vicente Alonso, Luís Antón Aparicio, Paula Jiménez Fonseca, Enrique Grande, Juan José Reina, José Luís Manzano, Juan Domingo Alonso Lájara, Jorge Barriuso, Daniel Castellano, Javier Medina, Carlos López, Ángel Segura, Sergio Carrera, Guillermo Crespo, José Fuster, Javier Munarriz, Pilar García Alfonso

**Affiliations:** 1 Medical Oncology Department, Vall d’Hebron University Hospital, Autonomous University of Barcelona, P. Vall d’Hebron 119-129, 08035 Barcelona, Spain; 2 Medical Oncology Department, Virgen de la Victoria University Hospital, Campus Universitario Teatinos, 29010 Málaga, Spain; 3 Medical Oncology Department, Miguel Servet University Hospital, Paseo Isabel la Católica, 1-3, 50009 Zaragoza, Spain; 4 Medical Oncology Department, University Hospital Complex, As Xubias, 84, 15006 A Coruña, Spain; 5 Medical Oncology Department, Asturias Central University Hospital, Calle Celestino Villamil, 33006 Oviedo, Spain; 6 Medical Oncology Department, Ramón y Cajal University Hospital, Ctra. de Colmenar Viejo, km. 9100, 28034 Madrid, Spain; 7 Medical Oncology Department, Virgen Macarena University Hospital, Avda Dr Fedriani, 3 41009 Sevilla, Spain; 8 Medical Oncology Department, Catalan Oncology Institute (ICO-Badalona), Germans Trias i Pujol University Hospital, Carretera Canyet s/n, Badalona, 08016 Barcelona Spain; 9 Medical Oncology Department, Virgen de la Arrixaca University Hospital, Ctra. Madrid Cartagena, 30120 Murcia, Spain; 10 Medical Oncology Department, La Paz University Hospital, Paseo de la Castellana 261, 28046 Madrid, Spain; 11 Medical Oncology Department, 12 de Octubre University Hospital, Avda. de Córdoba, 28041 Madrid, Spain; 12 Medical Oncology Department, Toledo Hospital Complex, Av de Barber 30, 45071 Toledo, Spain; 13 Medical Oncology Department, Marqués de Valdecilla University Hospital, Av Valdecilla, 39008 Santander, Spain; 14 Medical Oncology Department, La Fe University Hospital, Avinguda de Campanar 21, 46026 Valencia, Spain; 15 Medical Oncology Department, Cruces University Hospital, Plaza Cruces, 48903 Barakaldo, Vizcaya Spain; 16 Medical Oncology Department, Burgos University Hospital, Avda. Islas Baleares 3, 09006 Burgos, Spain; 17 Medical Oncology Department, Son Dureta University Hospital, C/ Andrea Doria 55, 07014 Palma de Mallorca, Spain; 18 Medical Oncology Department, Castellón Provincial Hospital Consortium, Av Doctor Clara 19, 12002 Castellón de la Plana, Spain; 19 Medical Oncology Department, Gregorio Marañon Hospital, Calle Doctor Esquerdo 46, 28007 Madrid, Spain

**Keywords:** Lanreotide, Neuroendocrine tumours, Sunitinib, Everolimus, Somatostatin analogues, Clinical practice, Cross-sectional analysis, Combination treatment

## Abstract

**Background:**

Based on the mechanism of action, combining somatostatin analogues (SSAs) with mTOR inhibitors or antiangiogenic agents may provide synergistic effects for the treatment of patients with neuroendocrine tumours (NETs). Herein, we investigate the use of these treatment combinations in clinical practice.

**Methods:**

This retrospective cross-sectional analysis of patients with NETs treated with the SSA lanreotide and targeted therapies at 35 Spanish hospitals evaluated the efficacy and safety of lanreotide treatment combinations in clinical practice. The data of 159 treatment combinations with lanreotide in 133 patients was retrospectively collected.

**Results:**

Of the 133 patients, with a median age of 59.4 (16–83) years, 70 (52.6 %) patients were male, 64 (48.1 %) had pancreatic NET, 23 (17.3 %) had ECOG PS ≥2, 41 (30.8 %) had functioning tumours, 63 (47.7 %) underwent surgery of the primary tumour, 45 (33.8 %) had received prior chemotherapy, and 115 (86.5 %) had received prior SSAs. 115 patients received 1 lanreotide treatment combination and 18 patients received between 2 and 5 combinations. Lanreotide was mainly administered in combination with everolimus (73 combinations) or sunitinib (61 combinations). The probability of being progression-free was 78.5 % (6 months), 68.6 % (12 months) and 57.0 % (18 months) for patients who only received everolimus plus lanreotide (n = 57) and 89.3 % (6 months), 73.0 % (12 months), and 67.4 % (18 months) for patients who only received sunitinib and lanreotide (n = 50). In patients who only received everolimus plus lanreotide the median time-to-progression from the initiation of lanreotide combination treatment was 25.8 months (95 % CI, 11.3, 40.3) and it had not yet been reached among the subgroup of patients only receiving sunitinib plus lanreotide. The safety profile of the combination treatment was comparable to that of the targeted agent alone.

**Conclusions:**

The combination of lanreotide and targeted therapies, mainly everolimus and sunitinib, is widely used in clinical practice without unexpected toxicities and suggests efficacy that should be explored in randomized prospective clinical trials.

## Background

Neuroendocrine tumours (NETs) are a heterogeneous group of relatively rare malignancies originating from the diffuse neuroendocrine system found most often in the bronchial or gastrointestinal systems [1]. Somatostatin analogues (SSAs) are a key therapeutic option in the management of advanced NETs, leading to a significant improvement in patient quality of life [2–5]. There are currently 2 SSAs in clinical use: octreotide [6] and lanreotide [7, 8]. Longer acting (slow-release and depot) formulations of SSAs include octreotide long-acting release (LAR), lanreotide Autogel and lanreotide LP. Small studies have suggested that treatment with SSAs is associated with disease stabilization and prolonged progression-free survival (PFS) in some patients with NETs [8, 9]. Moreover, following the randomized PROMID study confirming that octreotide delayed time to tumour progression (TTP) (from 6 to 14.3 months, hazard ratio [HR] = 0.34; p ≤ 0.0001) in patients with metastatic NETs [9], SSAs have been administered to patients to provide not only hormonal symptom control but also antitumour activity [10].

A Phase II trial carried out by the Spanish TTD group evaluated the efficacy of lanreotide Autogel 120 mg on tumour growth stabilization in 30 patients with progressive gastroenteropancreatic and bronchopulmonary NETs. The median PFS was 12.9 months with clinical benefit reported in 93 % of the patients [11]. In the international Phase III Clarinet trial lanreotide substantially prolonged PFS compared with placebo (HR = 0.47; 95 % CI 0.30–0.73; p < 0.001) in patients with non-functioning gastroenteropancreatic NETs [12].

Recent therapeutic advances with everolimus, a mammalian target of rapamycin (mTOR) inhibitor, and sunitinib, a multitargeted agent with antiangiogenic activity, have led to an improvement in patients with advanced pancreatic NETs (pNETs) [13–16]. Everolimus has shown antitumour activity in 2 Phase III studies (RADIANT-2 and RADIANT-3). In RADIANT-2, treatment with everolimus plus octreotide resulted in a 5.1-month increase in median PFS compared with placebo plus octreotide (16.4 *vs.* 11.3 months) in patients with advanced NETs with carcinoid syndrome, although the difference did not reach statistical significance [13]. In RADIANT-3, patients with progressive pNETs had a statistically significant improvement in PFS associated with everolimus compared with placebo (11 *vs.* 4.6 months). A Phase III study of sunitinib in patients with progressive pNETs was unblinded early after more than a doubling of median PFS (11.4 *vs.* 5.5 months) favoured the patients receiving sunitinib *vs.* placebo [14]. After a 2-year follow-up, the median overall survival (OS) was estimated at 33 months in the sunitinib arm [17].

The combination of SSAs and targeted therapies is a potential treatment option for patients with NETs [18]. Indeed, several small studies suggest that the combined use of octreotide and everolimus could provide an increase in efficacy [13, 19, 20]. Unfortunately, no randomized data have compared the outcome of patients who received a novel targeted agent alone *vs.* the combination with a SSA. However, in clinical practice, targeted therapies are frequently combined with SSAs and there have been reports of valuable efficacy in heavily pretreated patients [21]; thus in a retrospective cohort, 83 % of 29 patients with well differentiated pNETs who were treated with sunitinib in daily practice in Spain also received concomitant treatment with SSAs [22]. Furthermore, Barriuso *et al.*, reported that 87.5 % of 40 patients with NETs on treatment with sunitinib as palliative treatment in 6 Spanish hospitals, concomitantly received SSAs [23].

The aim of this retrospective cross-sectional analysis was to define the efficacy and safety of the SSA, lanreotide, in combination with antiangiogenic targeted therapies or inhibitors of the mTOR pathway in the routine clinical practice, to help evaluate their potential clinical benefit in the management of patients with NETs in Spain.

## Methods

### Design

Between July 2011 and October 2011 we collected the data from patient medical charts to perform a retrospective multicentre cross-sectional analysis of patients with NETs that were treated with the SSA lanreotide combined with novel targeted therapies. Data were collected from medical oncology services of Spanish hospitals with experience in the treatment of NETs with lanreotide and newer therapeutic agents, such as mTOR inhibitors or antiangiogenic agents (tyrosine kinase inhibitors [TKIs] or monoclonal antibodies). Thirty-five centres distributed over 27 Spanish provinces were identified and invited to participate in the project. The conduct of this retrospective cross-sectional analysis was approved by the ethics committee of the Vall d’Hebron University Hospital.

### Objectives

We wanted to determine the epidemiologic characteristics of the patients analysed, in terms of proliferative rate and location of the primary tumour, functionality, differentiation and tumour extension, as well as treatments received prior to the combination therapy. The main efficacy objectives included determining the drugs used in the course of the combined therapy, the length of this combination therapy, biochemical response (50 % reduction of chromogranin A), the radiologic response rate obtained according to Response Evaluation Criteria In Solid Tumours (RECIST) v1.0, and response duration. The radiologic images were not centrally reviewed by the investigators; the information on progression was obtained from the patients’ medical chart. TTP was defined as the time from the initiation of lanreotide combination therapy until there was an indication of disease progression as noted in the patients’ clinical history. In line with the retrospective nature of this analysis, it is important to point out that the progression status had no planning dates for the estimation of TPP. OS was defined from the initiation of lanreotide combination therapy until patient death. Safety objectives were to collect the reasons for discontinuing the combined therapy, and to define the adverse events (AEs) profile according to the Common Terminology Criteria for Adverse Events (CTCAE) v3.0.

### Patient population

All patients diagnosed with NET being followed at the medical oncology services who had received treatment with lanreotide in combination with a novel therapeutic target agent for at least 3 months prior to data collection into an electronic Data Report Form were eligible to be included in the retrospective analysis. All patients had progressed on previous treatment before receiving combination treatment with lanreotide. If the length of combination treatment did not reach 3 months, the patient would still be eligible for inclusion as long as treatment discontinuation was due to an AE. The 3-month minimum combined treatment cut-off would be used to exclude patients who abandoned combination treatment very early; however, there were patients included in the analysis who received combined treatment for less than 3 months. Upon progression with the lanreotide combination, patients received further treatment according to the standard of care at each centre.

### Statistical analysis

Summary statistics are presented for all variables. Efficacy was assessed on the basis of tumour response. Kaplan-Meier methods were used to obtain estimates of median TTP and OS, with corresponding HRs and 2-sided 95 % confidence intervals (CIs). The protocol was approved by the Ethics Committee of the hospitals where data was collected.

## Results

### Patient population

One hundred and thirty-three patients with a diagnosis of NET who received combination treatment with lanreotide and targeted therapy in the setting of routine clinical practice were analysed. Patients began receiving lanreotide combination treatment between April 2008 and July 2011. The demographic and clinical characteristics of the patients are described in Table 1. The median age of patients with NETs was 59.4 years, and their main comorbidities were hypertension and diabetes. Approximately half of the patients had pNETs; the primary site was the ileum in 21 (15.8 %) patients and the lung in 12 (9.0 %) patients. Almost all patients had metastatic disease at diagnosis (98.5 %) and the liver was the most common metastatic location (84.2 %). Thirty-one percent of patients had functional tumours (carcinoid, gastrinoma, somatostinoma and vasoactive intestinal peptide secreting tumour [VIP]oma). The majority of patients had received prior pharmacologic treatment. The number of prior treatment lines was 1 for 52 (39.1 %) patients, 2 for 31 (23.3 %) patients, 3 for 19 (14.3 %) patients, 4 for 9 (6.8 %) patients, and 5 for 6 (4.5 %) patients.

**Table 1:** Patient demographics, disease characteristics, and prior treatment regimens

Characteristic	Number of patients
	(N = 133)
Sex, n (%)	
Male	70 (52.6)
Female	63 (47.4)
Age, years	
Median (range)	59.4 (16–83)
Comorbidities, n (%)	
Hypertension	51 (38.3)
Diabetes	37 (27.8)
Dyslipidaemia	25 (18.8)
Heart disease	19 (14.3)
Liver disease	6 (4.5)
Hypothyroidism	12 (9.0)
Tumour extension at diagnosis, n (%)	
Locally advanced	6 (4.5)
Metastatic	127 (95.5)
Tumour extension at treatment initiation, n (%)	
Locally advanced	2 (1.5)
Metastatic	131 (98.5)
ECOG PS, n (%)	
0	45 (33.8)
1	65 (48.9)
2	22 (16.5)
3	1 (0.8)
Location of primary tumour, n (%)	
Foregut	85 (64.0)
Lung	12 (9.0)
Oesophagus	1 (0.8)
Stomach	3 (2.3)
Duodenum	5 (3.8)
Pancreas	64 (48.1)
Midgut	30 (22.6)
Jejunum	3 (2.3)
Ileum	21 (15.8)
Appendix	2 (1.5)
Cecum	4 (3.0)
Hindgut	6 (4.5)
Colon	3 (2.3)
Rectum	3 (2.3)
Unknown	12 (9.0)
Histological differentiation, n (%)	
Grade 1	55 (41.4)
Grade 2	42 (31.6)
Grade 3	2 (1.5)
Unknown	34 (25.6)
Location of metastases, n (%)	
Liver	112 (84.2)
Bone	18 (13.5)
Peritoneum	19 (14.3)
Lung	19 (14.3)
Lymph node	29 (21.8)
Other^a^	4 (3.0)
Tumour functionality, n (%)	
Non-functioning	92 (69.2)
Functioning	41 (30.8)
Carcinoid	13 (9.8)
Gastrinoma	1 (0.8)
Somatostinoma	1 (0.8)
VIPoma	3 (2.3)
Not specified	23 (17.3)
Ki-67 index, n (%)	
0–2	52 (39.1)
3–10	29 (21.8)
11–20	8 (6.0)
> 20	1 (0.8)
Unknown	43 (32.3)
Previous non-pharmacologic treatments, n (%)	
Surgery of the primary tumour	63 (47.4 %)
Surgery of metastases	21 (15.8 %)
Local treatments^b^	23 (17.3)
Radiotherapy	6 (4.5)
Previous pharmacologic treatments, n (%)	
None	16 (12.0)
Chemotherapy	45 (33.8)
Targeted therapy	16 (12.0)
Immunotherapy	7 (5.3)
Somatostatin analogues	
Lanreotide	78 (58.6)
Octreotide	37 (27.8)
Combination with somatostatin analogues	
Interferon	19 (14.3)
Targeted therapy	7 (5.3)

^a^Other metastatic sites include breast (n = 1), pleura (n = 1), spleen (n = 1), adrenal gland (n = 1)

^b^Includes embolization, (transarterial) chemoembolization, radiofrequency ablation and radioembolization

*ECOG PS*, Eastern Cooperative Group Oncology Performance Status; *VIPoma*, Vasoactive intestinal peptide secreting tumour

### Treatment and patient disposition

According to the investigators’ criteria, the main reason for combining lanreotide with targeted therapies was to achieve antiproliferative synergy (113 patients, 85.0 %). In the other patients, the main reason was to control hormonal symptoms. The majority (115 patients, 86.5 %) of patients received only 1 lanreotide treatment combination; but, overall, the 133 patients included in the analysis received a total of 159 combinations of targeted therapy with lanreotide (Table 2) so there were patients that received 2 or more combinations. As expected, the most common combinations were with everolimus (73 combinations, 45.9 % of the 159 combinations) and sunitinib (61 combinations, 38.4 % of the 159 combinations). Other combinations with targeted agents included bevacizumab (n = 9), sorafenib (n = 8), and pazopanib (n = 8); however, due to the small number of patients that received each of these combinations individual characterization of the outcomes of these combinations was not carried out. With a median follow-up of 43.9 months (range 1.8–628.7), the median duration of treatment was 5.1 months (range 0–35.6) in the 115 patients who only received 1 treatment combination. In the 57 patients who only received everolimus plus lanreotide the median follow-up was 42.2 months (range 1.8–275.1) and the median duration of treatment was 4.7 months (range 0–35.6). Similarly, in the 50 patients who only received the combination of sunitinib with lanreotide, the median follow-up was 31.8 months (range 2.8–628.7), with a median duration of treatment of 5.9 months (range 0.4-25.0). There were 4 patients receiving everolimus plus lanreotide (range 0.59–2.95 months) and 2 patients receiving sunitinib plus lanreotide (range 0.39–2.98 months) that received treatment for less than 3 months and discontinued due to an AE. In addition there were 5 patients receiving the everolimus and lanreotide combination for less than 3 months that discontinued due to tumour progression (range 0–2.98 months).

**Table 2:** Treatment combinations in the 133 patients analysed

	Number of patients
	(N = 133)
Number of treatment combinations, n (%)	
1	115 (86.0)
2	12 (9.0)
3	5 (3.8)
5	1 (0.8)
	**Number of treatment combinations**
	**(N = 159)**
Targeted agent combined with lanreotide, n (%)	
Everolimus	73 (45.9)
Sunitinib	61 (38.4)
Treatment discontinuation, n (%)^a^	84 (52.8)
Everolimus	n = 73
All discontinuations	39 (53.4)
Disease progression	23 (31.5)
Adverse event	10 (13.7)
Other	6 (8.2)
Ongoing	34 (46.6)
Sunitinib	n = 61
All discontinuations	27 (44.3)
Disease progression	15 (24.6)
Adverse event	10 (16.4)
Other	2 (3.3)
Ongoing	34 (55.7)

^a^The denominator is the number of treatment combinations with a each targeted agent

In 128 of the 159 combinations the dose of lanreotide Autogel was 120 mg every 28 days. Everolimus was administered at a dose of 10 mg/day in 72 combinations and at 5 mg/day in 1 combination. The administration of sunitinib was less homogeneous, 49 combinations with a continuous dose of 37.5 mg/day, 11 combinations of 50 mg/day sunitinib on a 4 weeks on/2 weeks off schedule, and 1 patient who received 25 mg/day.

At the time of the data cut-off, 84 treatment combinations (52.8 % of 159) had been discontinued. The reasons for treatment discontinuation were disease progression in 47 (29.6 % of 159) combinations and AEs in 24 (15.1 % of 159) combinations (Table 2).

Data on follow-up treatment was collected for 30 patients. Fourteen patients received a SSA, either as monotherapy or in combination with another agent. Five patients received sunitinib, either as monotherapy or in combination with a SSA and four patients received everolimus, either as monotherapy or in combination with lanreotide. Eight patients received chemotherapy combinations.

### Efficacy

Overall, 23 treatment combinations led to a tumour response (1 complete response [CR] and 22 partial responses [PRs]) resulting in an objective response rate of 14.5 % with all treatment combinations (Table 3). Stable disease (SD) was reported in 113 (71.1 %) treatment combinations and the disease control rate was 85.5 %. The response (18.3 %; with 1 CR and 20 PRs) and disease control rates (82.6 %) were similar in the subgroup of the 115 patients who only received 1 treatment combination. Chromogranin A expression was measured in 37 (27.8 %) patients, with normalization reported in 6 (16.2 % of 37) patients and a reduction in 10 (27.0 % of 37) patients. A correlation between chromogranin A expression and radiologic tumour response was not carried out because many chromogranin A measurements were missing. One third of the patients did not have Ki67 data and proliferation index was not analysed.

**Table 3:** Radiologic response rate in all 133 patients (analysed all 159 treatment combinations) and in the 115 patients that only received one lanreotide combination

All patients	All treatment combinations	Everolimus + lanreotide	Sunitinib + lanreotide
	**N = 159** ^**a**^	**n = 73**	**n = 61**
Tumour response (%)			
Complete response	1 (0.6)	0	1 (1.6)
Partial response	22 (13.8)	11 (15.1)	9 (14.8)
Stable disease	113 (71.1)	49 (67.0)	42 (68.9)
Progressive disease	13 (8.2)	9 (12.3)	3 (4.9)
Not evaluated	10 (6.2)	4 (5.5)	6 (9.8)
**Patients that received only one lanreotide combination**	**One treatment combination**		
	**n = 115**	**n = 57**	**n = 50**
Tumour response (%)			
Complete response	1 (0.9)	0	1 (2.0)
Partial response	20 (17.4)	10 (17.5)	8 (16)
Stable disease	74 (64.3)	35 (61.5)	34 (68.0)
Progressive disease	11 (9.6)	8 (14.0)	2 (4.0)
Not evaluated	9 (7.8)	4 (7.0)	5 (10)

^a^The denominator is the total number of treatment combinations in 133 patients analysed

### Subanalysis of patients that only received everolimus and lanreotide or sunitinib and lanreotide

**Fig. 1:**
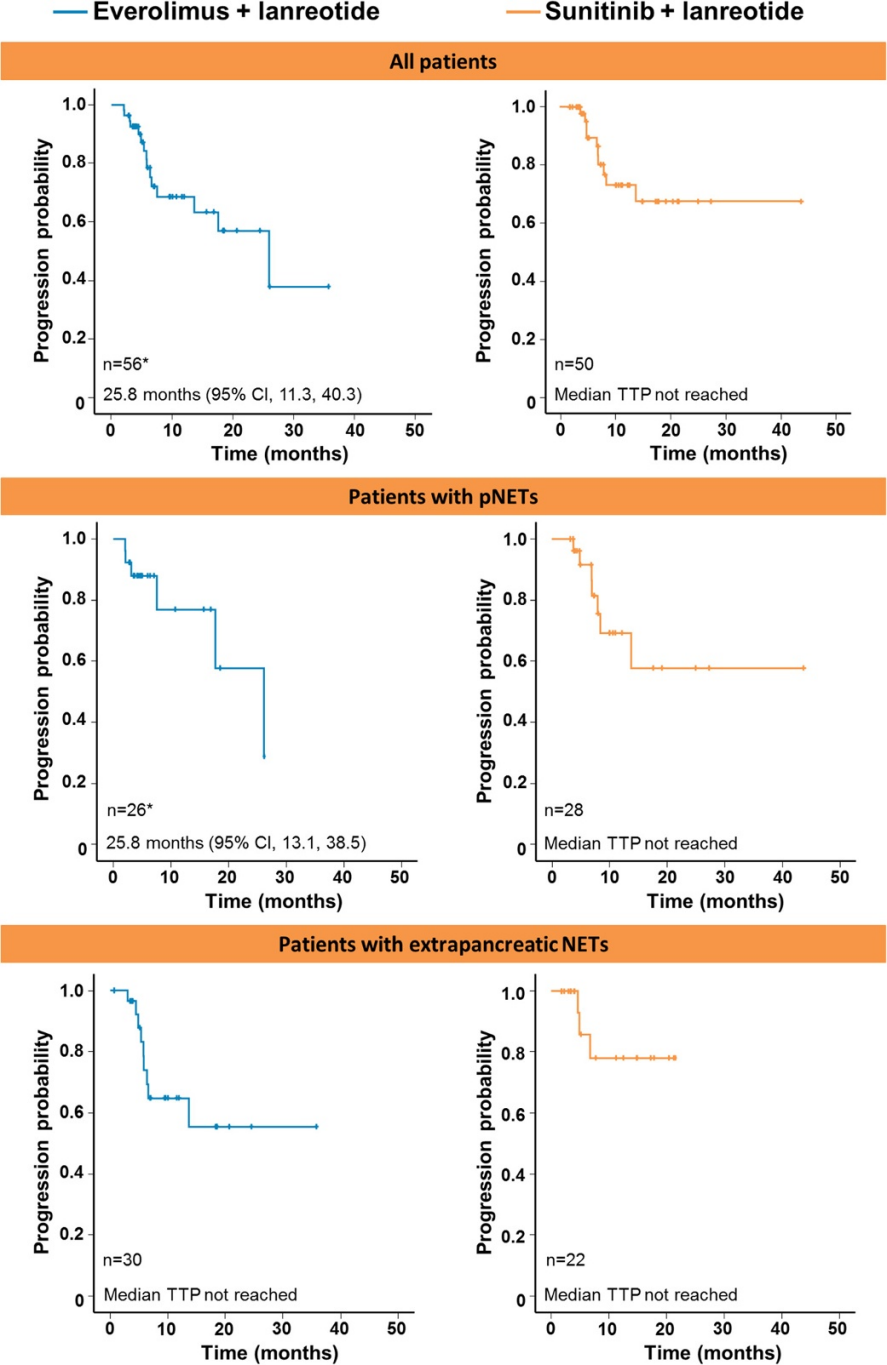
Time to progression. Kaplan-Meier curves indicating the time to progression in all patients receiving only everolimus + lanreotide (n = 56)* or sunitinib + lanreotide (n = 50), in patients with pNETs receiving everolimus + lanreotide (n = 26)* or sunitinib + lanreotide (n = 28), and in patients with extrapancreatic neuroendocrine tumours receiving everolimus + lanreotide (n = 30) or sunitinib + lanreotide (n = 22). *Information on tumour progression was missing in 1 patient receiving everolimus + lanreotide

**Fig. 2:**
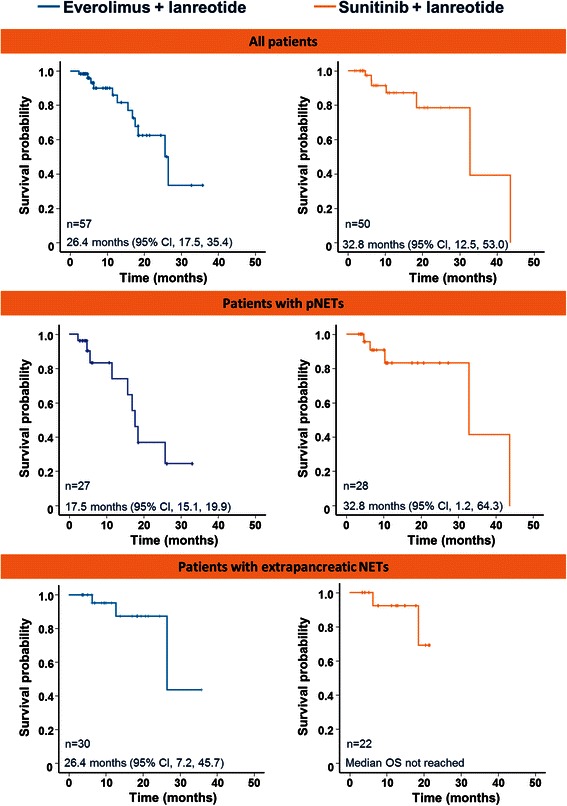
Overall survival. Overall survival Kaplan-Meier curves in all patients receiving only everolimus + lanreotide (n = 57) or sunitinib + lanreotide (n = 50), in patients with pNETs receiving everolimus + lanreotide (n = 27) or sunitinib + lanreotide (n = 28), and in patients with extrapancreatic neuroendocrine tumours receiving everolimus + lanreotide (n = 30) or sunitinib + lanreotide (n = 22)

Among the 115 patients who received only 1 lanreotide treatment combination, 57 patients received everolimus plus lanreotide and 50 patients received sunitinib plus lanreotide. In patients who only received everolimus plus lanreotide the median TTP from the initiation of combination treatment was 25.8 months (95 % CI, 11.3, 40.3) and it had not yet been reached among the subgroup of patients receiving sunitinib and lanreotide (Fig. 1). The probability of being progression-free at 6 months was 78.5 % in the everolimus and lanreotide group and 89.3 % in the sunitinib and lanreotide group and at 12 months it was 68.6 and 73.0 %, in the everolimus and sunitinib patient groups, respectively. At 18 months, 57.0 % of patients receiving everolimus plus lanreotide and 67.4 % of patients receiving sunitinib plus lanreotide were estimated to be free of progression. The median OS was 26.4 months (95 % CI, 17.5, 35.4) for patients receiving everolimus and lanreotide and 32.8 months (95 % CI, 12.5, 53.0) for sunitinib subgroup (Fig. 2).

### Safety

Overall there were 270 AEs in 97 patients (Table 4). The majority of AEs reported were Grade 1 (n = 115) or 2 (n = 106) in severity, with few Grade 3 (n = 39) or Grade 4 (n = 9) AEs. Generally, the safety profile of the combination with lanreotide resembled the safety profile of the targeted agent in monotherapy (Table 5). The main AEs were asthenia, mucositis, and diarrhoea. There were 6 AEs (5 AEs were Grade 1 or 2) that were related to lanreotide administration; these included diarrhoea, hyperglycaemia, and abdominal pain.

**Table 4:** Treatment-related AEs in all patients; N = 133

	All AEs	Grade ≥ 3
	n (%)	n (%)
Asthenia	48 (36.1)	13 (9.8)
Mucositis	35 (26.3)	6 (4.5)
Diarrhoea	33 (24.8)	4 (3.0)
Hand-foot skin reaction	20 (15.0)	1 (0.8)
Anorexia	16 (12.0)	2 (1.5)
Hyperglycaemia	13 (9.8)	1 (0.8)
Rash	12 (9.0)	3 (2.3)
Hypertension	12 (9.0)	1 (0.8)
Peripheral oedema	7 (5.3)	2 (1.5)
Thrombocytopenia	7 (5.3)	3 (2.3)
Anaemia	7 (5.3)	1 (0.8)
Pneumonitis	4 (3.0)	1 (0.8)
Cardiac toxicity	4 (3.0)	3 (2.3)
Hypercholesterolemia	4 (3.0)	0
Hypertriglyceridemia	4 (3.0)	0
Leucopoenia	4 (3.0)	1 (0.8)
Hepatic alterations	3 (2.3)	0
Hypothyroidism	3 (2.3)	0
Nausea	3 (2.3)	0
Vomiting	3 (2.3)	0
Abdominal pain	3 (2.3)	0
Weight loss	3 (2.3)	0
Headache	1 (0.8)	0
Epistaxis	1 (0.8)	0
Other	20 (15.0)	6 (4.5)

AE = adverse event

**Table 5:** Number of adverse events (AEs) and Grade 3 or 4 AEs reported during the study and assignment of causality to the treatment received. The number of Grade 3 and 4 AEs is shown in parenthesis

	Everolimus	Lanreotide and everolimus	Sunitinib	Lanreotide and sunitinib	Lanreotide
	All AEs (Grade 3–4)	All AEs (Grade 3–4)	All AEs (Grade 3–4)	All AEs (Grade 3–4)	All AEs (Grade 3–4)
AE	129 (21)	9 (2)	70 (17)	15 (5)	6 (1)
Asthenia	15 (5)	0	22 (8)	2 (0)	0
Mucositis	25 (6)	0	6 (0)	0	0
Diarrhoea	17 (2)	1 (0)	5 (1)	5 (1)	1 (0)
Hand-foot skin reaction	8 (0)	0	8 (1)	0	0
Anorexia	9 (1)	0	4 (1)	0	0
Hyperglycaemia	7 (0)	4 (1)	0	1 (0)	1 (0)
Rash	10 (2)	1 (1)	0	0	0
Hypertension	2 (0)	0	6 (1)	0	0
Peripheral oedema	6 (2)	0	1 (0)	0	0
Thrombocytopenia	0	0	4 (0)	3 (3)	0
Anaemia	5 (0)	0	0	2 (1)	0
Pneumonitis	3 (1)	0	1 (0)	0	0
Cardiac toxicity	0	0	3 (2)	0	0
Hypercholesterolemia	4 (0)	0	0	0	0
Hypertriglyceridemia	4 (0)	0	0	0	0
Leucopoenia	1 (0)	0	2 (1)	1 (0)	0
Hepatic alterations	0	2 (0)	0	0	0
Hypothyroidism	0	0	3 (0)	0	0
Nausea	1 (0)	0	0	0	0
Vomiting	1 (0)	0	1 (0)	0	0
Abdominal pain	1 (0)	0	0	0	2 (0)
Weight loss	1 (0)	0	0	1 (0)	0
Headache	0	0	0	0	0
Epistaxis	0	0	1 (0)	0	0
Other	9 (2)	1 (0)	3 (2)	0	2 (1)

At the data cut-off, 3 patients were alive without disease, 106 patients were alive with disease and there had been 24 deaths (22 due to disease progression, 1 cardiac insufficiency and 1 death in a patient receiving everolimus plus lanreotide that was not due to disease progression and was potentially considered by the investigator to be a Grade 5 AE).

## Discussion

This cross-sectional analysis retrospectively evaluated the clinical use of the SSA lanreotide in combination with targeted agents in Spanish patients with advanced NETs in the setting of routine clinical practice. As expected, in the majority of patients, lanreotide was administered with everolimus or sunitinib. The probability of being progression-free was encouraging in the patient population analysed (patients who survived or maintained treatment for more than 3 months). The estimated proportion of patients who were alive and progression-free at 18 months was 34 % with everolimus in the RADIANT-3 trial [15] and in the sunitinib Phase 3 trial it was estimated that 71.3 % of patients were alive and progression-free at 6 months [14].

In the RADIANT-3 trial there were 40 % of patients that received concomitant treatment with SSAs, but median PFS for treatment with everolimus was similar in the group of patients that received SSAs (11.4 months) and in the group of patients that did not (10.8 months) [24]. In the Phase II RADIANT-1 study, the median PFS by central radiology review was 16.7 months and the median OS had not been reached at the time of data cut-off in the subgroup of patients who received everolimus plus octreotide [19]. In the subgroup of patients receiving everolimus monotherapy median PFS was 9.7 months and median OS was 24.9 months. A subanalysis of the 40 % of patients receiving SSAs in the Phase III sunitinib study showed that their use resulted in a nonstatistically significant improvement in PFS (HR 0.78; p = 0.31) compared with the patients who received no on-study SSA [25].

In our cross-sectional analysis there might appear to be differences in the efficacy results between everolimus or sunitinib; however, this analysis was not set up to compare the data between the different targeted agents that are routinely combined with lanreotide in clinical practice and therefore it should not be assumed that one of the targeted agents analysed here would be a better combination partner for lanreotide. There are several limitations that should be taken into account when dissecting the data in our cohort of patients. This is a cross-sectional and retrospective analysis of patients being treated at selected sites that are presumed to be reference sites for the treatment of NETs and to have experience in the management of novel targeted agents. Furthermore, there was a bias in the selection process since the patients included in this retrospective analysis should have been receiving treatment for at least 3 months except for those who did not tolerate the combination. This inherent selection bias probably underestimates the number of patients in clinical practice with early progression with the combination strategy. There were no strict timelines to assess tumour response, no central review of the images, and patient follow up was performed according to local guidelines. In addition, the sample of the analysis is very heterogeneous since there are several patients who received subsequent lines of treatment, including maintenance with lanreotide alone. Furthermore, the dose of sunitinib that patients received was heterogeneous; the majority of patients received continuous daily dosing (the schedule that is approved in Europe for patients with pNETs), but a considerable share of patients followed the intermittent 4 weeks on and 2 weeks off schedule that is the approved schedule for advanced renal cell carcinoma (RCC) and gastrointestinal stromal tumour (GIST) [26]. In addition to taking these limitations into account, it is important to highlight that any potential clinical benefits should be confirmed in studies specifically designed to evaluate whether combination therapy with a SSA is superior to the targeted agent alone. Several trials are currently ongoing: SUNLAND (ClinicalTrials.gov NCT01731925) is a clinical trial aimed at evaluating the activity of sunitinib, alone or in combination with lanreotide, in midgut carcinoids. In addition, a randomized phase II study, COOPERATE-2 (ClinicalTrials.gov NCT01374451), evaluating the treatment effect of everolimus in combination with the SSA pasireotide relative to everolimus alone on PFS in patients with advanced progressive pNET, has completed accrual. Furthermore, LUNA (ClinicalTrials.gov NCT01563354) will test the effectiveness and safety of everolimus or pasireotide alone or in combination in adult patients with advanced neuroendocrine carcinoma (typical and atypical) of the lung and thymus. The results from these studies are eagerly awaited.

Combination of lanreotide with targeted therapies did not lead to a significant increase in AEs when compared with the safety profile of each targeted agent as monotherapy. Most common AEs of SSA treatment are usually mild, limited in time, and can include local reactions (pain and erythema) at the injection site, abdominal cramps, nausea, flatulence, diarrhoea, steatorrhoea and a risk of cholelithiasis, more common after long exposure to the drug [2].

## Conclusions

The combination of lanreotide and everolimus or sunitinib is widely used in routine clinical practice at Spanish hospitals without unexpected toxicities. The median TTP of the patients receiving the combined treatment with lanreotide appears to be clinically relevant. Furthermore, the data suggest that the combination of lanreotide and everolimus or sunitinib might provide tumour control in the majority of patients with NETs receiving treatment. The possibility of enhanced efficacy when combining SSAs and targeted therapies, suggests that this approach should be further explored in randomized prospective clinical trials.

## Abbreviations

AE: Adverse event
CI: Confidence intervals
CR: Complete response
CTCAE: Common Terminology Criteria for Adverse Events
ECOG PS: Eastern Cooperative Group Oncology Performance Status
HR: Hazard ratio
mTOR: mammalian target of rapamycin
NETs: Neuroendocrine tumours
OS: Overall survival
pNETs: Pancreatic neuroendocrine tumours
PFS: Progression-free survival
PR: Partial response
RECIST: Response Evaluation Criteria In Solid Tumours
SD: Stable disease
SSA: Somatostatin analogues
TKI: Tyrosine kinase inhibitor
TTP: Time to tumour progression
VIPoma: Vasoactive intestinal peptide secreting tumour
